# Mitophagy in ototoxicity

**DOI:** 10.3389/fncel.2023.1140916

**Published:** 2023-02-23

**Authors:** Hezhou Han, Sainan Hu, Yue Hu, Dongliang Liu, Junbo Zhou, Xiaofang Liu, Xiulan Ma, Yaodong Dong

**Affiliations:** ^1^Department of Otolaryngology Head and Neck Surgery, Shengjing Hospital of China Medical University, Shenyang, China; ^2^Institute of Medical Genetics and Applied Genomics, University of Tübingen, Tübingen, Germany; ^3^Department of Surgical Oncology, The First Affiliated Hospital of China Medical University, Shenyang, China

**Keywords:** mitophagy, ototoxicity, mitochondria, PINK1-Parkin, autophagy receptors

## Abstract

Mitochondrial dysfunction is associated with ototoxicity, which is caused by external factors. Mitophagy plays a key role in maintaining mitochondrial homeostasis and function and is regulated by a series of key mitophagy regulatory proteins and signaling pathways. The results of ototoxicity models indicate the importance of this process in the etiology of ototoxicity. A number of recent investigations of the control of cell fate by mitophagy have enhanced our understanding of the mechanisms by which mitophagy regulates ototoxicity and other hearing-related diseases, providing opportunities for targeting mitochondria to treat ototoxicity.

## Introduction

Hair cells in the inner ear are specialized sensory cells which transmit sound and balance information to the central nervous system. Hair cell dysfunction or even death can be easily caused by the use of certain drugs, overexposure to various environmental stimuli (e.g., noise, toxic chemicals), as well as aging (Steyger, 2021) and leads to further sensorineural hearing loss. These ototoxic factors are regarded as mainly damaging hair cells by inducing mitochondrial dysfunction, as the cochlea has high energy requirements. Although hair cells do not have the highest metabolic rate relative to other inner ear structures, such as the stria vascularis and supporting cells (Nakai and Hilding, 1968; Marcus et al., 1978), they are more susceptible to mitochondrial oxidative stress, due to their lower antioxidant content (Sha et al., 2001).

Mitochondria provide energy to the cells (Cheng et al., 2010). In addition to their role as energy producers, mitochondria play key roles in many other cellular processes required to maintain cellular homeostasis, including the regulation of calcium stores and initiation of apoptosis. They also serve as scaffolds in cellular metabolic events, including iron-sulfur cluster biogenesis, amino acid synthesis, and lipid metabolism (Aoun and Tiranti, 2015; Bhola and Letai, 2016). Thus, the maintenance of healthy mitochondria is critical for cell survival and proper functioning. Although this process is critical for cell fate, the inevitable generation of reactive oxygen species (ROS) damages proteins and lipids, and—at elevated levels—can damage the mitochondria (Schieber and Chandel, 2014). In addition to ROS, misfolded proteins and mutant mitochondrial DNA (mtDNA) also induce damage, and mitochondria respond to minor damage through a series of mechanisms, such as controlling the fusion/fission cycle or the mitochondrial unfolded protein response (UPR^mt^) (Roberts et al., 2016). Owing to the central role of mitochondria in cellular homeostasis, it seems self-evident that their proper function is critical for maintaining auditory function, and, indeed, many pathways are involved in the maintenance of mitochondrial integrity.

Mitochondrial dysfunction in ototoxicity has been discussed in many studies (Selimoglu, 2007; Op de Beeck et al., 2011). Some ototoxic agents, such as aminoglycoside-targeting bacteria, also readily damage the mitochondria within cochlear hair cells, resulting in the release of pro-apoptotic factors and oxidative enzymes into the cytoplasm and, consequently, the generation of free radicals (Priuska and Schacht, 1995; Sha and Schacht, 1999; Warchol, 2010). As for noise-induced hearing loss, immediately after noise overstimulation, the level of free Ca^2+^ in outer hair cells increases (Fridberger et al., 1998), and elevated Ca^2+^ has been shown to significantly stimulate mitochondrial ROS production in neurons and other cells, resulting in cell death (Peng and Jou, 2010). ROS, mitochondrial DNA mutations, and mitochondrial apoptosis all contribute to age-related hearing loss. Previous studies have indicated that ROS and other dysfunctions of the mitochondria serve as triggers for cellular defense pathways, including autophagy (Vernon and Tang, 2013). Autophagy is an elaborate degradation and recycling mechanism that breaks down unnecessary or dysfunctional cellular components through a highly regulated process in all eukaryotic cells (Kobayashi, 2015). However, autophagy has contradictory functions in cells. On the one hand, overactivation of autophagy can promote myocardial cell death during ischemia/reperfusion and overactivation of autophagy can also promote the occurrence of pathological changes, as is the case in liver fibrosis (Clarke, 1990; Yu et al., 2004; Ryter et al., 2014). On the other hand, autophagy can remove damaged organelles, defend against microbial infection, and has a protective effect against metabolic, heart disease, and neurodegenerative diseases (including Parkinson’s, Alzheimer’s, and Huntington’s) (Yu et al., 2004). In hair cells, autophagy plays an important role in preventing ototoxicity, including aminoglycoside-, cisplatin-, noise-, and age-induced hearing loss (Liu et al., 2021). Based on these findings, targeting autophagy to control mitochondrial quality appears to be a potential strategy to attenuate ototoxicity. It has been increasingly recognized that autophagy is specifically regulated in highly differentiated cells, with clear tissue-specific differences associated with autophagy regulation. For example, Mizushima et al. (2004) used a transgenic mouse model expressing the autophagosomal marker green fluorescent protein-microtubule associated protein 1 light chain 3 (GFP-LC3) to demonstrate that autophagy is significantly upregulated upon starvation in liver tissue but not in the brain. Differentiated and highly polarized cells, such as neurons, exhibit spatially separated autophagy and mitophagy pathways, which were recently shown to be critical for the control of mitochondrial homeostasis and regulation of cellular function (Maday and Holzbaur, 2014; Sung et al., 2016).

Mitophagy, meaning the selective degradation of mitochondria, was first proposed in 2005 (Lemasters, 2005). It is a basic cellular mechanism, evolutionarily conserved from yeast to humans, that regulates cell quality and homeostasis (Onishi et al., 2021). Multiple signaling events are involved in the execution of mitophagy, including (a) mitochondrial fission from the network, (b) targeted mitochondria marking mediated by ubiquitin-dependent or independent mitophagy receptors, (c) phagophore recruitment and expansion, mitochondrial engulfment by autophagosomes (mature phagophores), and (d) fusion of autophagosome and a lysosome to form the autolysosome for final cell degradation (Montava-Garriga and Ganley, 2020). Mitophagy is a multifactorial and intricate process, and dysfunction at any step is likely to cause mitophagy discontinuity.

Dysfunction of mitophagy is particularly relevant in neurodegenerative disease (Rodolfo et al., 2018). The proteins phosphatase and tensin homolog-induced kinase 1 (PINK1) and Parkin, which are genetic factors in Parkinson’s disease, play a protective role in neurons through mitophagy (Ashrafi et al., 2014). During the progression of Alzheimer’s disease, Parkin is involved in the removal of mitochondria (Ye et al., 2015) and its overexpression can alleviate the symptoms of the disease (Martín-Maestro et al., 2016). Mitophagy is also altered in Huntington’s disease. The mutant huntingtin acts as a scaffold to induce selective autophagy (Martinez-Vicente et al., 2010) or negatively affects mitochondrial delivery to the lysosomes (Wong and Holzbaur, 2014). The results of recent studies have indicated that mitophagy can also contribute to ototoxicity. Increasing mitophagy levels can protect hair cells from aminoglycoside-induced damage (Zhang et al., 2022). Mitophagy levels were shown to decrease in mice with age-related hearing loss (AHRL), and overexpression of dynamin-related protein 1 (DRP1) improved the mitochondrial function of HEI-OC1 cells (Lin et al., 2019). Although the current study seems to demonstrate the regulation of hair cell function by mitophagy, the specific function of mitophagy remains inconclusive. In this review, we focus on the regulatory function of mitophagy in ototoxicity and related signaling networks. In addition, the possible targets that actively regulate mitophagy to protect hair cells from damage are discussed.

## Dysfunction of mitochondria in ototoxicity

Mitochondria are the targets of ototoxicity for multiple reasons. Mitochondria have their own genomes that encode mitochondrial proteins. It has been well-established that mtDNA has a higher mutation rate than nuclear DNA does, and that repair mechanisms are weaker in mtDNA than in nuclear DNA. Therefore, mitochondria play a very important role in cell survival (Wallace et al., 1987). The A1555G mutation in the mitochondrial genome is associated with an increased risk of aminoglycoside ototoxicity. The mutation is in the gene encoding mitochondrial 12S rRNA. By replacing adenine at position 1,555 with guanine, guanine forms an additional base pair with cytosine at position 1,494 in the 12S rRNA ternary structure. This makes the mutated 12S rRNA more similar to bacterial 16S rRNA, which is a key target of aminoglycosides (Prezant et al., 1993). Besides, the 745A > G, 792C > T, 839A > G, 856A > G, 1310C > T, and 1452T > C variants located in the 12S rRNA stem also have a similar mechanism to A1555G mutation, reported to be associated with aminoglycoside antibiotics toxicity (Lu et al., 2010). In addition, ROS are considered to be involved in aminoglycoside-induced ototoxicity. Aminoglycosides have been reported to cause hearing loss by generating free radicals in the inner ear and subsequently damaging sensory cells and neurons (Rybak and Kelly, 2003). ROS are normal products of the metabolic processes of organisms. It functions as a regulatory messenger in diverse processes such as proliferation, survival, gene expression, and apoptosis (Kopke et al., 1999). Under normal circumstances, ROS are easily cleared by antioxidants such as catalase, superoxide dismutase (SOD), and glutathione in the body, preventing ROS from escaping into the cells, thereby maintaining the homeostasis of the inner ear. Aminoglycosides, however, can combine with transition metals, such as iron and copper, to form free radicals. It has been reported that the Fe II-aminoglycoside complex can bind to phosphatidylinositol and induce the release of arachidonic acid (Dehne et al., 2002). Simultaneously, arachidonic acid can form a ternary complex with iron and aminoglycosides, leading to the formation of ROS (Lesniak et al., 2005). Excess ROS damage to the mitochondria has also been observed in cisplatin-induced ototoxicity. Cisplatin causes direct DNA damage in cochlear hair cells. After cisplatin uptake by outer hair cells, water molecules or water ligands replace the chlorinated ligands on cisplatin to form hydrates. Hydrated cisplatin enters the nucleus and binds to the guanine bases of DNA, creating DNA crosslinks and disrupting DNA repair. During DNA damage, superoxide anion (O _2_^–^), a type of ROS, are generated, leading to hair cell apoptosis (Wang et al., 2023). Mitochondria have also been found to be important sites for age-induced ototoxicity. In the cochlea, it has been hypothesized that cochlear mitochondrial redox imbalance and mtDNA mutations and deletions may be synergistically involved in ARHL (Chen and Tang, 2014). A specific common mitochondrial deletion (mtDNA4977) was frequently observed in the temporal bones of ARHL patients, and its measured level was closely related to the severity of ARHL (Markaryan et al., 2009). Both deletions and mutations of mtDNA are increased in the cochlea of ARHL patients compared to normal hearing subjects (Bai et al., 1997; Fischel-Ghodsian et al., 1997). Furthermore, reduced mitochondrial complex IV activity has been observed postmortem in temporal spiral ganglion neurons from aged ARHL patients (Markaryan et al., 2010). In noise-induced hearing loss (NIHL), noise exposure results in oxidative stress, calcium overload, and energy metabolism disturbances, which are intimately linked to mitochondrial dysfunction. Different types of ototoxicity cause mitochondrial damage in the inner ear through diverse mechanisms, and various degrees of exposure to external factors result in disparate cellular responses. In some cases, the cell can compensate for mitochondrial damage and refresh its mitochondrial network, whereas in other cases, the cell will clear the damaged mitochondria by mitophagy or cell death will occur.

## Mitophagy maintain mitochondrial homeostasis

Mitochondria in cells with high energy demand are often numerous, forming an expansive dynamic functional and structural network that requires certain mechanisms to maintain the homeostasis of the mitochondrial network. The balance between mitochondrial biogenesis and mitophagy is the crux of mitochondrial homeostasis (Chen and Tang, 2014). Mitochondrial biogenesis is defined as the generation or renewal of healthy mitochondria, whereas mitophagy involves the removal of damaged or superfluous mitochondria (Cardanho-Ramos and Morais, 2021). This is a dynamic process that is closely related to mitochondrial fission and fusion, involving DRP1, mitofusion 1 and 2 (MFN1/2), optic atrophy 1 (OPA1), mitochondrial fission factor (MFF), and peroxisome proliferator-activated receptor gamma coactivator-1α (PGC-1α).

Upon minor mitochondrial damage, a series of mechanisms are initiated to rescue the targeted mitochondria, including, but not limited to, DNA repair, proteolysis, UPR^mt^ and fusing damaged mitochondria with healthy mitochondria to share components. UPR^mt^ is a cellular stress response that alleviates mitochondrial proteotoxicity by upregulating mitochondrial chaperones and proteases (Pickles et al., 2018). However, when mitochondria are severely damaged, if fusion cannot rescue the damaged mitochondria, the damaged components or the whole mitochondria undergo fission from the healthy mitochondrial network and are eliminated *via* mitophagy (Youle and van der Bliek, 2012). Mitophagy is a double-edged sword that can promote cell survival and accelerate cell apoptosis, depending on physiological and pathological context (Joaquim and Escobar-Henriques, 2020). Under appropriate stress, upregulation of mitophagy can remove damaged mitochondria and prevent apoptosis; however, overwhelming mitophagy may ultimately result in cell death due to inadequate healthy mitochondria to maintain function (Cho et al., 2021).

## Process of mitophagy

**FIGURE 1:**
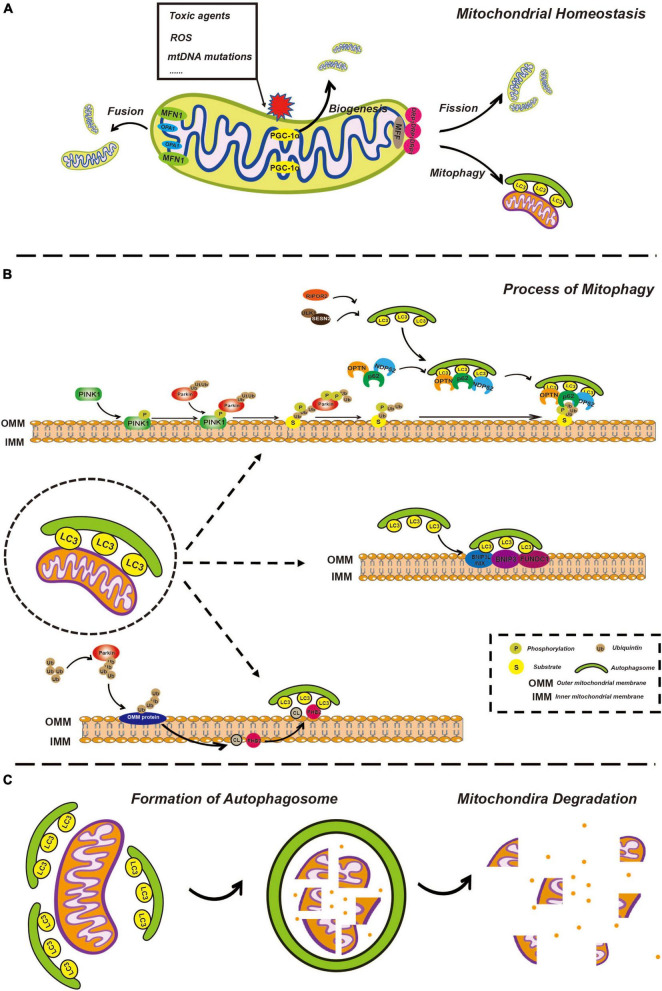
Overview of mitophagy. **(A)** Overview of mitochondria fusion, fission, and biogenesis. Healthy mitochondria constantly undergo fusion and fission processes, allowing for the exchange of genetic material and the maintenance of optimal mitochondrial shape and function. Fusion of two or more mitochondria results in a larger, more complex structure through the action of fusion proteins such as mitofusins (MFN1 and MFN2) and opticatrophy 1 (OPA1). Fission of a single mitochondrion into smaller, individual units helps to distribute energy evenly throughout the cell and is mediated by the fission protein, dynamin-related protein 1 (DRP1). Mitochondrial biogenesis involves the creation of new mitochondria through the process of replication and division of existing mitochondria, facilitated by the peroxisome proliferator-activated receptor gamma coactivator-1α (PGC-1α) and other biogenesis factors, ensuring that energy production is maintained in the face of changes in cellular demand. **(B)** The process of mitophagy is regulated by phosphatase and tensin homolog-induced kinase 1 (PINK1)/Parkin-dependent and independent pathways. In the PINK1/Parkin-dependent pathway, damaged mitochondria are recognized by the presence of the kinase protein PINK1. PINK1 activates the E3 ubiquitin ligase Parkin, which targets the damaged mitochondria for degradation by attaching ubiquitin molecules to the damaged mitochondria. The ubiquitin-tagged mitochondria are then enveloped by autophagosomes and delivered to the lysosome for degradation by lysosomal enzymes. In the PINK1/Parkin-independent pathway, damaged mitochondria are recognized by other factors, such as BCL2 interacting protein 3-like (BNIP3L)/NIX, cardiolipin (CL), and prohibitin 2 (PHB2) which then trigger autophagosome formation and subsequent delivery to the lysosome for degradation. **(C)** Mitochondria degeneration. Autophagosome formation is regulated by a series of autophagy-related proteins, such as microtubule associated protein 1 light chain 3 (LC3). Once the autophagosome has formed, it fuses with a lysosome, allowing for the degradation of the enclosed mitochondria by lysosomal enzymes, including cathepsins. The lysosomal enzymes break down the enclosed mitochondria into its constituent parts, which can then be recycled or eliminated from the cell.

In mammalian cells, under some stressful conditions, damaged mitochondria undergo membrane potential loss, isolation, or fission from the network, relying on DRP1 activity or autophagy machinery (Montava-Garriga and Ganley, 2020). Mitochondrial fragmentation due to imbalanced fission and fusion of mitochondria is a prerequisite of mitophagy (Shirihai et al., 2015; Chen et al., 2016). Mitochondrial fission in mammalian cells is primarily mediated by DRP1, a member of the cytosolic dynamin family (Youle and van der Bliek, 2012). In mammals, fusion between the OMM is mediated by membrane-anchored dynamin family members named MFN1 and MFN2, whereas fusion between the IMM is mediated by a single dynamin family member called OPA1 (Youle and van der Bliek, 2012; Figure 1A). Later, isolated mitochondria might be marked through different pathways of mitophagy involving ubiquitin-dependent or ubiquitin-independent mitophagy receptors.

In most mammalian cells, the PINK1-Parkin pathway is the most extensively characterized mechanism for regulating mitophagy. PINK1 is a mitochondrial serine/threonine-protein kinase with a kinase domain and mitochondrial localization sequence (Geisler et al., 2010; Lazarou et al., 2012; Li Y. et al., 2022). Parkin is an E3-ubiquitin ligase (Gatica et al., 2018) responsible for the specific recognition and ubiquitination of target proteins. In different situations, this pathway acts in different ways. On the one hand, newly synthesized PINK1 in the cytoplasm is continuously targeted to mitochondria with normal membrane potential by the mitochondrial localization sequence, enters the inner mitochondrial membrane (IMM) through the translocase of the outer membrane (TOM) and translocase of the inner membrane (TIM) (Kerr et al., 2017) and is cleaved by the inner membrane protease presenilin-associated rhomboid-like (PARL) (Jin et al., 2010; Deas et al., 2011). PARL is a critical regulator of mitochondrial homeostasis through the cleavage of its substrates and functions in mitochondrial quality control and apoptosis (Arutyunova et al., 2021). Processed PINK1 is rapidly degraded through the proteasome-dependent pathway as soon as it is released from the mitochondrial membrane space (IMS) into the cytoplasm.

In contrast, damaged mitochondria undergo depolarization of the mitochondrial membrane potential, which subsequently inhibits IMM insertion and subsequent cleavage of PINK1, leading to accumulation of PINK1 on the outer mitochondrial membrane (OMM) (Jin et al., 2010; Meissner et al., 2011; Pickles et al., 2018). PINK1 activates ubiquitin through phosphorylation, and ubiquitin binds to a variety of substrates that are localized on the OMM in a process called substrate ubiquitination. Likewise, PINK1 recruits Parkin from the cytosol and phosphorylates Parkin to partially activate Parkin-enhancing E3 ligase activity, so that more ubiquitin binds to substrates on OMM to form ubiquitin chains (Lazarou et al., 2015). PINK1 phosphorylates ubiquitin chains more efficiently, while ubiquitin can further activate Parkin, thus forming a positive feedforward loop (Pickles et al., 2018). PINK1 is believed to release the “eat me” signal and Parkin is assumed to act as a signal amplifier.

Subsequently, many autophagy receptors, including sequestosome-1 (p62), optineurin (OPTN), nuclear domain 10 protein 52 (NDP52), TAX1 binding protein 1 (TAX1BP1), and BRCA1 adjacent gene 1 (NBR1), are recruited from the cytoplasm to damaged mitochondria and interact with ubiquitinated proteins in OMM. In addition to autophagy receptors recruited by the PINK1-Parkin pathway, several specific receptors localized on the OMM also respond to mitochondrial damage, such as BCL2 interacting protein 3-like (BNIP3L)/NIX, BNIP3, and FUN14 domain containing 1 (FUNDC1) (Kim et al., 2021). Moreover, prohibitin 2 (PHB2), and cardiolipin (CL), which are located on the IMM, are reported to initiate mitophagy following the process of outer mitochondrial membrane-associated protein degradation (OMMAD) (Karbowski and Youle, 2011; Palikaras et al., 2018). OMMAD refers to the proteasome-mediated degradation of ubiquitylated OMM proteins, such as MFN1/2, resulting in OMM rupture and exposure of IMM receptors to the cytoplasm.

Except for sequential signal events occurring on the mitochondria itself, the formation of the phagophore is gradual. The phagophore is a bilayer membrane structure and the precursor of the autophagosome, which is derived from the endoplasmic reticulum, Golgi apparatus, and plasma membrane (Zhen et al., 2020; Gudmundsson et al., 2022). The phagosome expands, extends, curves, and develops from a flat bilayer membrane to a large circular structure. Simultaneously, the phagophore is gradually transported to and surrounds the damaged mitochondria and eventually engulfs the target mitochondria. It goes without saying that this process requires a variety of autophagy-related (Atg) proteins and complexes and we will discuss some of them, particularly which would be mentioned in further detail related to ototoxicity below. UNC-51-like autophagy-activating kinase 1 (ULK1) plays a key role in autophagy initiation. Once autophagy begins, the ULK1 complex is recruited to the phagophore, which in turn recruits other multiprotein complexes such as the Atg8/LC3/gamma-aminobutyric acid receptor-associated protein (GABARAP) complex (Montava-Garriga and Ganley, 2020). Atgs are a series of proteins encoded by autophagy-associated genes. LC3, a classic autophagic marker, is common in general autophagy pathways and is synthesized in the pro-LC3 form, cleaved by ATG4B to form LC3-I, and conjugated to phosphatidylethanolamine (PE) to form LC3-II (Ashrafi and Schwarz, 2013). Upon binding to the protein complex, an LC3-positive phagophore is formed, which gradually aggregates around damaged mitochondria. LC3 is an important bridge that connects autophagosomes and mitochondria. The autophagy receptors mentioned above possess an LC3 interacting region (LIR) that is recognized by and interacts with phagophores *via* LIR (Lazarou et al., 2015; Figure 1B).

After engulfment of the mitochondria, the phagophore closes, becoming a spherical autophagosome. The autophagosome then fuses with a lysosome, turning into the autolysosome, while the cargo, specifically the damaged mitochondria, is degraded, thus completing the renewal and sustaining the functional stability of the mitochondria. It is worth mentioning that whether these are chronological events or occur simultaneously remains unclear. For example, mitochondrial fission from the network can be driven prior to the formation of a phagophore; however, the forming phagophore can also directly mediate mitochondrial isolation (Montava-Garriga and Ganley, 2020; Figure 1C).

In the following section, we summarize and discuss the changes and roles of mitophagy-related molecules in ototoxicity, regardless of the temporal sequence of mitophagy.

## Mitochondrial fission, fusion, and biogenesis in ototoxicity

Mitochondrial dynamics are critical in regulating morphology, number, subcellular distribution, and function. This process can be seen in Figure 1A. In brief, mitochondrial fission and fusion are tightly regulated by a complex protein machinery involving DRP1, MFN1, MFN2, and OPA1 in mammalian cells. Mitochondrial fission was found to contribute to mitochondrial apoptosis and is also thought to be a prerequisite for mitophagy, while mitochondrial fusion is associated with increased mitochondrial metabolism (Chen et al., 2016).

Given the importance of DRP1 in mitophagy, the role of DRP1 in ototoxicity has attracted the attention of researchers. Lin et al. (2019) established a cellular senescence model of HEI-OC1cells and cochlear explants from C57BL/6 mice, which are known for their early onset hearing loss. The authors discovered that the inhibition of DRP1 by Mdivi-1 blocked mitophagy and exacerbated hearing loss in aged C57BL/6 mice. Mdivi-1 is a targeted inhibitor of DRP1, which can inhibit mitochondrial fission by reducing the activity of DRP1, attenuate OMM permeabilization, and reduce the rate of mitochondria-mediated apoptosis (Manczak et al., 2019; Nhu et al., 2021). In recent studies, Mdivi-1 has emerged as a promising therapeutic compound that reduces mitochondrial dysfunction and apoptosis in myocardial infarction and neurodegenerative diseases (Manczak et al., 2019; Nhu et al., 2021). According to the results reported by Lin et al. (2019), Mdivi-1 does harm hearing, and DRP1-dependent mitochondrial fission might promote mitophagy and alleviate hearing loss in aged mice. Furthermore, the expression of DRP1 was observed at the protein level rather than at the gene level, and mitophagy levels were evaluated by LC3 and p62, which are not exclusive markers of mitophagy but are shared with general autophagy. On the contrary, Mdivi-1 is expected to increase viability of hair cells against cisplatin toxicity in a zebrafish model (Vargo et al., 2017). In another study, Yamashita and Kanki (2017) monitored time-lapse imaging of mitophagy and showed that Dnm1 (homolog of DRP1) like-independent mitochondrial division occurs concomitantly with autophagosome formation, which calls into question the necessity of DRP1 in mitophagy. Thus, the role of DRP1 in mitophagy needs to be more precisely addressed. In summary, mitophagy might be an effective way for hair cells to survive aging stress, and inhibition of DRP1 may block mitophagy and aggravate ARHL. However, mitophagy is believed to be initiated in a number of ways, and whether Mdivi-1 is absolutely deleterious to hearing or may also become beneficial depending on the circumstances remains to be elucidated.

As mentioned above, MFN2 is an important mitochondrial fusion factor, and MFF is a mitochondrial fission factor that promotes mitochondrial division by recruiting DRP1, both of which may have important effects on mitophagy. In a study of hearing and mitophagy in aged mice, significant upregulation of MFN2 and downregulation of MFF were observed, accompanied by an increased level of mitophagy. As pointed out by Kim et al. (2021), whether these particular genes could be research targets in the future treatment of ototoxicity still needs to be explored in more focused experiments.

Peroxisome proliferator-activated receptor gamma coactivator-1α is a master regulator of mitochondrial biogenesis and is highly expressed in tissues with high-energy demands (Rius-Pérez et al., 2020). PGC-1α is essential for controlling the expression of genes associated with anti-ROS and the electron transport chain complex (Wu et al., 2019). PGC-1α exhibits age-related decrease and may be an important contributing factor to mitochondrial function in age-related diseases (Chen and Tang, 2014); for example, the overexpression of PGC-1α significantly decreased the accumulation of damaged mtDNA and cell apoptosis in the strial marginal cells senescence model (Zhao et al., 2013). PGC1-α levels have also been found to be increased in hair cellss and the auditory cortex, which may enhance sensitivity to ARHL (Feng et al., 2020).

Studies have shown that PGC-1α plays a protective role in noise-induced, age-related, and drug-induced hearing loss by reducing the rate of hair cell apoptosis (Zhao et al., 2013; Xue et al., 2016; Hao et al., 2019; Han et al., 2020; Liu et al., 2022). Chen et al. (2020) established a animal model of guinea pigs for the pathogenesis of NIHL and their findings suggest that ginsenoside Rd ameliorates NIHL by activating the SIRT1/PGC-1α signaling pathway, which can be an attractive pharmacological target for the development of novel drugs for NIHL treatment. Another study supports a link between age-related ototoxicity and miR-29b/SIRT1/PGC-1α signaling. The authors found that miR-29b overexpression in HEI-OC1 cells by transfection with miR-29b mimic inhibited SIRT1 and PGC-1α expression, leading to an increase in mitochondrial dysfunction and cochlea hair cell apoptosis. Moreover, the inhibition of miR-29b by transfection with miR-29b inhibitor increased SIRT1 and PGC-1α expression, rescuing hair cell apoptosis (Xue et al., 2016). Although PGC-1α-related signaling pathways have been shown to play diverse roles in protecting various types of hearing loss, however, whether these pathways ultimately affect ototoxicity by regulating mitochondrial biogenesis is still unclear, and the mechanisms of PGC-1α in ototoxicity are expected to be investigated in more detail.

There are many proteins indispensable for mitochondrial fission and fusion, but their roles in mitophagy remain unclear. Given that they have not been explored in related ototoxicity studies, we will not mention them here in detail. It is expected that more experiments will be conducted in the future, which will not be limited to *in vitro* cell lines.

## PINK1-Parkin pathway participates in ototoxicity

Noise exposure, ototoxic drugs, aging, and genetic mutations all contribute to ototoxicity. Among the various forms of ototoxicity, oxidative stress is a key determinant of the fate of cochlear cells, which is pivotal in mitophagy. As upstream regulators of mitophagy, PINK1 and Parkin are important for the removal of damaged mitochondria or their components and the maintenance of normal mitochondrial turnover. Stabilization of the mitochondrial respiratory chain (RC), composed of oxidative phosphorylation (OXPHOS) complexes, appears to ensure ATP production. The loss of PINK1 or Parkin has been shown to delay protein turnover in OXPHOS complexes *via* impaired mitophagy (Vincow et al., 2013). Failure of damaged RC subunits to degrade may contribute to cell senescence and neurodegeneration (Pickles et al., 2018). Considering that PINK1 and Parkin are the core proteins in mitophagy, and their potential function in ototoxicity is of utmost importance, researchers are exploring relevant molecular mechanisms through various methods. To date, morphological and functional changes in the mitochondria and expression of PINK1 and Parkin have been observed in induced ototoxicity in different models.

Studies have shown that the expression of PINK1 and Parkin decreases in the mouse auditory cortex and inferior colliculus with age, indicating that mitophagy may contribute to the cellular changes observed in an aged central auditory system, which results in age-related ototoxicity (Oh et al., 2020; Youn et al., 2020). Increased expression of PINK1 and Parkin was observed in aged C57BL/6 mice after long-term supplementation with the natural antioxidant resveratrol and PINK1-Parkin-dependent mitophagy significantly prevented hair cell loss, spiral ganglion neuron loss, and stria vascular atrophy in aged C57BL/6 mice (Xiong et al., 2019). Resveratrol is a SIRT1 activator, and SIRT1 is an NAD-dependent deacetylase that participates in a broad range of biological activities, including cell cycle, response to DNA damage, metabolism, aging, apoptosis and autophagy (Imperatore et al., 2017; Alves-Fernandes and Jasiulionis, 2019; Xu et al., 2020). It is reasonable to assume that, in C57BL/6 mice, SIRT1 overexpression alleviates age-related cochlear hair cell loss and hearing loss through activation of the PINK1-Parkin pathway to ease the cellular survival burden due to oxidative stress. Therefore, SIRT1 is expected to be a novel target for regulating mitophagy in hair cells.

In another study, it was claimed that the presence of Parkin might fulfill the anti-aging activity of urolithin A (UA), a mitophagy activator commonly used in mammalian cells. Using premature senescent auditory cells [H_2_O_2_-induced aging HEI-OC1 cells, which are one of the few, and arguably the most commonly used mouse auditory cell line available for research purposes (Kalinec et al., 2016)] and cochlear explants, Cho et al. (2022) found that UA treatment significantly reduced the expression of p53 and p21 and increased the expression of mitophagy-related proteins, ATP content, mtDNA integrity, and mitochondrial membrane potential, while the knockdown of Parkin was able to abrogate the function of UA. p53 and p21 are classical markers of cellular senescence that induce senescence by promoting cell cycle arrest (Regulski, 2017; Mijit et al., 2020). This suggests that UA may resist cell senescence and apoptosis through Parkin-dependent mitophagy, which reduces the accumulation of damaged mitochondria and increases ATP production, promoting cell survival (Cho et al., 2022).

Drug ototoxicity limits the quality of life of patients after treatment. More than 150 drugs are currently known to be ototoxic, including aminoglycosides, glycopeptides, macrolide antibiotics, platinum-based anticancer drugs, loop diuretics, quinine, and salicylate analgesics (Lanvers-Kaminsky et al., 2017). Different drugs presumably cause ototoxicity through various mechanisms, including inhibition of mitochondrial protein biosynthesis and active ion transport, constriction of blood flow in the cochlea, increased ROS production in mitochondria, and decompensation of oxidative metabolism (Lanvers-Kaminsky et al., 2017; Steyger, 2021). Researchers have suggested that oxidative stress induces apoptosis and necrosis in hair cells of the cochlea, marginal cells, and stria vascularis (Lanvers-Kaminsky et al., 2017). Mitochondria are the main source of ROS and are responsible for the maintenance of normal inner ear function. According to studies, the PINK1-Parkin pathway may play a vital role in drug-induced ototoxicity. PINK1 alleviates gentamicin-induced ototoxicity mainly resulted from ROS by inducing mitophagy and resisting cell senescence and apoptosis in hair cells (Yang et al., 2018). PINK1 knockdown cells showed lower expression of LC3B, but a higher degree of p53 and activated caspase 3 in HEI-OC1 cells, and caspase 3 is the main executor of apoptosis and plays a crucial role in apoptosis (Lossi et al., 2018; Yang et al., 2018). Cisplatin ototoxicity is strongly assumed to be associated with oxidative stress and ROS production in cochlear cells aggravated by mitochondrial damage (Cho et al., 2021). Accordingly, the authors of that study then investigated the role of mitophagy in the regulation of cisplatin-induced ototoxicity using HEI-OC1 cells. The results showed that cisplatin gradually reduced the number of viable cells over time, induced mitochondrial depolarization, decreased intracellular ATP concentration, and enhanced the expression of PINK1, Parkin, and LC3 (Cho et al., 2021). In addition, siRNA-mediated parkin knockdown accelerated cisplatin-induced loss of cell viability, mitochondrial membrane potential, autophagosome/lysosome formation, and reduction in intracellular ATP production (Cho et al., 2021). However, the role of the PINK1-Parkin pathway in the regulation of mitophagy in drug-induced ototoxicity is inconsistent. In another study, researchers demonstrated that abolishing the expression of PINK1 or Parkin prevented the death of hair cells and subsequent hearing loss caused by the release of aminoglycosides (Li J. et al., 2022), indicating that mitophagy is likely to promote ototoxicity under certain circumstances. Nevertheless, there are also studies showing that neither neomycin nor gentamicin exposure has an impact on the level of mitophagy, suggesting a mitophagy-independent pathway of aminoglycoside ototoxicity (He et al., 2017; Setz et al., 2018).

In view of the limitations posed by technology and medical ethics, studies on the physiological and pathological mechanisms of drug-induced ototoxicity have been exclusively conducted *in vitro*. We cannot obtain more experimental data from animal models that can better correlate with the clinical manifestations of ototoxicity observed in humans (Lanvers-Kaminsky et al., 2017). In addition, although immortalized cells represent excellent model systems for investigating the spatiotemporal regulation of molecular pathways, they often are characterized by differences between humans and other animals that cannot be ignored (Moore and Holzbaur, 2016). With the progress of current studies on mitophagy in ototoxicity, more precise molecular targets and detailed interplay networks are expected to be detected.

## Mitophagy receptors in ototoxicity

### Autophagy receptors recruited from cytoplasm in ototoxicity

As mentioned above, the PINK1-Parkin pathway activates the polyubiquitination loop as soon as mitochondrial damage is sensed. After the ubiquitin chain is formed, autophagy adaptor proteins, including p62, OPTN, NDP52, NBR1, and TBX1BP1, are recruited to damaged mitochondria.

During autophagosome formation, p62 acts as a bridge between LC3 and polyubiquitinated proteins, which are selectively encapsulated in the autophagosome and then degraded by proteolytic enzymes in autolysosomes. Therefore, p62 protein expression is negatively correlated with autophagic activity. P62 is recognized as a classic marker of autophagy, which is not limited to mitophagy, and dramatic changes in p62 expression have been observed in studies of ototoxicity (He et al., 2017; Lin et al., 2019; Zhou et al., 2020).

Furthermore, NDP52 mediates mitophagy in neomycin-induced ototoxicity. Li W. et al. (2022) found that neomycin treatment induced mitophagy and decreased mitochondrial membrane potential in HEI-OC1 cells, resulting in increased ROS levels and apoptosis. Fasudil (Fas), a novel isoquinoline sulfonamide derivative, has been shown to have antioxidant capacity in previous studies and can inhibit neomycin-induced hair cell damage, which is achieved by negative regulation of NDP52 expression (Li W. et al., 2022). The authors speculated that excessive mitophagy inhibits the antioxidant capacity of Fas in NDP52 overexpressed cells, indicating that NDP52 aggravated neomycin-induced ototoxicity. Thus far, it is still unclear whether Fas could be a promising treatment target for ototoxicity and whether NDP52-involved mitophagy is harmful to hearing.

Optineurin is the most important adapter for the recruitment of phagophores to the mitochondria (Moore and Holzbaur, 2016). Specifically, Tank-binding kinase 1 (TBK1) can phosphorylate NDP52 and OPTN to enhance their activity (Richter et al., 2016), which is activated by the PINK1-parkin pathway after mitochondrial damage, which is also a feedforward mechanism (Heo et al., 2015). OPTN is a multifunctional autophagy receptor that participates in pathological changes in neurodegenerative disorders, such as amyotrophic lateral sclerosis and frontotemporal dementia (Lou et al., 2020). However, there is no clear evidence to show how OPTN plays a role in ototoxicity. Except for OPTN, TAX1BP1, and NBR1, which also exhibit important functions in mitophagy, require further study to investigate whether and how they affect ototoxicity.

### Autophagy receptors on OMM in ototoxicity

As mentioned above, BNIP3L/NIX, BNIP3, and FUNDC1, several specific receptors localized on OMM, also participate in the induction of mitophagy *via* direct interaction with LC3 (Kim et al., 2021).

BNIP3L/NIX was initially reported to restore mitochondrial sequestration from red blood cells during maturation (Sandoval et al., 2008). BNIP3L/NIX-mediated mitophagy is Parkin-independent and—as has been reported in studies of mice—plays a protective role in ischemic brain injury. Knockdown of both Parkin and BNIP3L/NIX synergistically reduces mitophagy (Yuan et al., 2017). In a recent study, researchers found that BNIP3L/NIX enhances mitophagy and synapse density in neurons and that BNIP3L/NIX activation may be a potential target for restoring synaptic function (Choi et al., 2021). Recently, Kim et al. (2021) found that BNIP3L/NIX maintains cochlear cell homeostasis during hearing aging by promoting mitophagy. The authors observed significant morphological changes in mitochondria in the cochlear tissue of aged mice, including cristae alterations, mitochondrial vacuolization, OMM rupture, and autophagosome increase, whereas most genes regulating mitophagy, including Parkin, PINK1, BNIP3, BNIP3L/NIX, and FUNDC1, were downregulated in an age-dependent manner. BNIP3L/NIX is expressed in Corti hair cells, supporting cells, spiral ganglion cells, and fibrocytes in the lateral wall; immunohistochemistry showed that BNIP3L/NIX levels in these cells were significantly decreased in aged mouse cochlea, accompanied by increased expression of TOMM20, p62, and LC3B, which indicates an accumulation of dysfunctional mitochondria and a lack of mitophagy (Kim et al., 2021). The expression of BNIP3L/NIX protein was detected in an *in vitro* cell senescence model induced by H_2_O_2_, and the results showed that BNIP3L/NIX may protect cochlear cells from senescence or apoptosis by reducing oxidative stress response and maintaining normal mitophagy (Kim et al., 2021). Likewise, decreased BNIP3 expression and damaged mitophagy were observed in the auditory cortex, inferior colliculus, and cochlea of aging mice and in HEI-OC1 cells treated with cisplatin, suggesting that BNIP3 activation plays a potential role in age- and cisplatin-induced ototoxicity (Oh et al., 2020; Youn et al., 2020; Cho et al., 2021).

FUN14 domain containing 1 is an OMM protein that mediates hypoxia-induced mitophagy in mammalian cells (Wu et al., 2016). The mitochondrially localized phosphoglycerate PGAM5 interacts with and dephosphorylates FUNDC1 under hypoxia, thus enhancing its interaction with LC3 (Chen et al., 2014). According to a study by Zhou et al. (2017), cardiac ischemia activated mitophagy by modifying FUNDC1 dephosphorylation, which substantively engulfed mitochondrial debris and cytochrome c, thus blocking apoptosis signal. It is also reported that FUNDC1-dependent mitophagy protects neurons against cerebral ischemia-reperfusion injury (Cai et al., 2021). However, it has also been reported that knockdown or overexpression of FUNDC1 has an insignificant effect on starvation- or hypoxia-induced mitophagy (Hirota et al., 2015); thus, the function of FUNDC1 is not clear. Similarly, in another study, there was no significant change in FUNDC1 expression after cisplatin exposure of HEI-OC1 cells, whereas other mitophagy-related markers showed a significant effect (Cho et al., 2021). In contrast, FUNDC1 was reported to be downregulated in aged cochlear tissue of mice. To date, it remains to be determined whether FUNDC1 participates in various forms of ototoxicity.

### Autophagy receptors on IMM in ototoxicity

Following OMM breakage, PHB2 interacts with the phagophore *via* its LIR motif (Wei et al., 2017; Yan et al., 2020). Yu et al. (2021) used C57BL/6 mice and the HEI-OC1 cell line as a model and found that PHB2 was expressed in hair cells, spiral ganglion neurons, and HEI-OC1 cells. The authors showed that, in ARHL mice, mitophagy was reduced and the corresponding PHB2 expression level was also reduced. After H_2_O_2_ treatment, mitophagy was activated and PHB2 expression increased, suggesting that PHB2-mediated mitophagy might mitigate age-induced ototoxicity against oxidative stress (Yu et al., 2021).

A recent mechanism of mitophagy initiation exploits lipids, instead of proteins, as mitophagy receptors. CL, a membrane lipid in IMM, can also function as an LC3 receptor in mitophagy (Yoo and Jung, 2018). Mitochondrial damage can result in the translocation of CL from the IMM to the OMM, or at least promote its exposure to the cytoplasm. These findings indicate that IMM components also participate in mitophagy; however, whether CL-mediated mitophagy influences ototoxicity remains to be determined.

### Formation of phagophore in ototoxicity

UNC-51-like autophagy-activating kinase 1 has been reported to be involved in noise-induced ototoxicity. Li Y. et al. (2022) established a noise-induced hearing loss model using C57BL/6J mice and HEI-OC1 cells treated with H_2_O_2_ and evaluated mitophagy activity by monitoring the expression of PINK1, Parkin, and LC3-II proteins. Sestrin 2 (SESN2), an endogenous antioxidant protein, interacts with ULK1, promoting ULK1 stabilization at the protein level and restoring ULK1 expression in SESN2-silenced cells, thus correcting mitophagy defects. The results of the 2022 study provide novel insights into SESN2 as a therapeutic target against noise-induced cochlear injury.

Recently, GABARAP was suggested to be a promising target for mitophagy in aminoglycoside-induced ototoxicity. Using HEI-OC1 cells and cochlear explants from mice, Li Y. et al. (2022) found that aminoglycosides bind to and trigger rapid translocation of Rho family interacting cell polarization regulator 2 (RIPOR2) in cochlear hair cells from the stereocilia to the pericuticular area, which interacts with the core autophagy component GABARAP and affects autophagy activation. Consequently, reducing the expression of RIPOR2 or GABARAP completely prevented aminoglycoside-induced hair cell death and subsequent hearing loss in mice (Li Y. et al., 2022). If these effects can be replicated and confirmed in further research, RIPOR2 and GABARAP are likely to be potential targets for the treatment of ototoxicity.

As mentioned previously, the limitations in current technologies—including the limitations of immortalized cells as simulations of human cell physiology—as well as the priority of medical ethics (which exclude humans as experimental subjects), prevent more in-depth study of ototoxicity.

### Targeting mitophagy as potential therapeutic interventions for ototoxicity

Considering about the role of mitophagy in ototoxicity, it seems reasonable to target mitophagy as therapeutic interventions for ototoxicity. In fact, according to current studies, some strategies have been discovered showing ability to regulate mitophagy in ototoxicity (We organized current reported agents/genomic interfere which can be found in Table 1). Because compared with gene intervention technology (siRNA, RNAi, plasmids, et, al), compounds or drugs that have been clinically applied may have a shorter distance from practical application. So we discuss some compounds or drugs that have been reported to alleviate ototoxicity by targeting mitophagy which might contribute to the development of specific drugs to prevent or treat ototoxicity.

**TABLE 1:** Summary of potential targets for treating ototoxicity.

Target	Ototoxicity type	Agents/Genomic interfere	Mechanism	Model system	References
PGC-1α	Noise-related ototoxicity	Ginsenoside Rd	Increase mitochondrial activity and reduce oxidative stress	Guinea pigs	Chen et al., 2020
PGC-1α	Age-related ototoxicity	MiR-29b inhibitor	Enhance mitochondrial function	HEI-OC1 cells	Xue et al., 2016
DRP1	Cisplatin-related ototoxicity	Mdivi-1	Reduce mitochondrial fission	Zebrafish	Vargo et al., 2017
PINK1 or Parkin	Age-related ototoxicity	Resveratrol	Improve the balance between mitophagy and mitochondrial biogenesis	HEI-OC1 cells Mice	Xiong et al., 2019
PINK1 or Parkin	Age-related ototoxicity	Urolithin A	Initiate mitophagy	HEI-OC1 cells Mice	Cho et al., 2022
NDP52	Neomycin-related ototoxicity	Fasudil	Inhibit mitophagy	HEI-OC1 cells	Li W. et al., 2022
BNIP3L/NIX	Age-related ototoxicity	Plasmid	Initiate mitophagy	HEI-OC1 cells Mice	Kim et al., 2021
ULK1	Noise-related ototoxicity	Sestrin 2	Initiate mitophagy	HEI-OC1 cells Mice	Li Y. et al., 2022

Ginseng is a well-known herbal medicine in China, which occupies a very important position in traditional Chinese medicine and has a long history of clinical application. Ginsenoside Rd, the active compound in ginseng, is known to have broad-spectrum pharmacological effects, reducing nerve damage that can lead to neurological disorders, including Alzheimer’s disease, Parkinson’s disease, Huntington’s disease, depression, cognitive impairment and cerebral ischemia (Chen et al., 2022). Ginsenoside Rd can ameliorate noise-induced auditory cortex damage by activating the SIRT1/PGC-1α signaling pathway, which may also be an attractive pharmacological target for the development of new drugs for noise-induced ototoxicity treatment (Chen et al., 2020). Similar to ginseng Rd, resveratrol, as a SIRT1 agonist, can improve the balance between mitophagy and mitochondrial biogenesis by activating the SIRT1/PGC1a signaling pathway, and significantly reduce age-related cochlear hair cell loss, Spiral ganglion neuron loss, stria vascular atrophy, and hearing threshold changes in C57BL/6 mice (Xiong et al., 2019).

Mdivi-1, as a DRP1 inhibitor can inhibit mitochondrial fission. Mdivi-1 was reported to protect hair cells of zebrafish lateral line neuromasts from cisplatin-induced ototoxicity (Vargo et al., 2017). But the role of Mdivi-1 in ototoxicity seems not unified. According to the result from another research, inhibition of DRP-1 by Mdivi-1 blocks mitophagy and exacerbates hearing loss in aged C57BL/6 mice Lin et al. (2019). While it is somehow explainable, because different models were used in the studies. More experiments are necessary to be conducted before Mdivi-1 can be used as the treating strategy for ototoxicity. Agents targeting the PINK1/Parkin pathway may have the ability to treat ototoxicity. Urolithin A induces mitophagy in various mammalian cells. Studies have shown that Urolithin A can significantly reduce the expression of senescence-associated p53 and p21 and increase the expression of mitophagy-related proteins in H_2_O_2_ -induced senescent cells and cochlear explants. Urolithin A is a natural compound, these complex polyphenols are found in foods such as pomegranates, berries and nuts. In recent years, it has been found that it has a very significant regulatory effect on nervous system diseases, so it can be considered as a drug for the treatment of ototoxicity (D’Amico et al., 2021).

The vast majority of research conclusions imply that mitophagy dysfunction is the main biological occurrence of ototoxicity, which can explain mitophagy is activated after hair cell damaged. Following this line, many studies focusing on promoting mitophagy as a protective strategy. While interestingly, another study found that Fas (a novel isoquinoline sulfonamide derivative, has exhibited antioxidant abilities in a number of previous studies) was able to prevent hair cell from neomycin-induced damage by inhibiting autophagy (Li W. et al., 2022). The results indicate mitophagy is complicated and dynamic and more evidence are necessary until targeting mitophagy as the therapeutic interventions for ototoxicity.

## Conclusion

Significant progress has been made in understanding the pathogenesis of ototoxicity. Mitochondrial dysfunction has been found to be an important feature of the pathological mechanism of ototoxicity, and mitophagy has also been implicated in regulating the occurrence and development of ototoxicity. Disruption of mitophagy has been observed in drug-, noise-, and aging-induced ototoxicity models. In addition, impairment of key proteins that promote mitophagy, such as PINK1-Parkin, can aggravate ototoxicity, which can be relieved by mitophagy agonists. Alternatively, changes in protein expression can attenuate ototoxic phenotypes. Considering the results of numerous studies in recent years, it is clear that mitophagy is an important process in ototoxicity. Further studies using various animal models of ototoxicity and possibly tissue samples of from ototoxic patient will aid in subsequent investigations of the molecular mechanism of impaired mitophagy, with the goal of developing new therapies to treat hearing loss.

## Author contributions

YD, XL, and XM designed this study. HH, SH, YH, JZ, and DL wrote the manuscript. All authors contributed to the article and approved the submitted version.
